# The Association between Two Common Polymorphisms in MicroRNAs and Hepatocellular Carcinoma Risk in Asian Population

**DOI:** 10.1371/journal.pone.0057012

**Published:** 2013-02-20

**Authors:** Miao Hu, Lianying Zhao, Surong Hu, Jingting Yang

**Affiliations:** 1 Department of Geriatrics, Changzhou NO 2 People's Hospital, Affiliated Hospital of Nanjing Medical University, Changzhou, China; 2 Kunshan Agency for Public Health Inspection, Soochow, China; University of North Carolina School of Medicine, United States of America

## Abstract

**Background:**

Emerging evidence has shown that microRNAs (miRNAs) participate in human carcinogenesis as tumor suppressors or oncogenes. Single nucleotide polymorphism (SNP) located in the miRNAs may influence the function of mature miRNAs and then affect the processing of carcinogenesis. It has been suggested that two common SNPs rs2910164 in miR-146a and rs3746444 in miR-499 are associated with susceptibility to hepatocellular carcinoma (HCC). However, published results are inconsistent and inconclusive. To acquire a more precise effect of the association between these polymorphisms and HCC risk, we performed this meta-analysis.

**Methodology/Principal Findings:**

We have conducted a search of case-control studies on the associations of SNPs rs2910164 and/or rs3746444 with susceptibility to HCC in PubMed, ScienceDirect, Cochrane Central Register of Controlled Trials, and Chinese National Knowledge Infrastructure databases for the period up to Sep 10th, 2012. A total of 6 studies were identified with 2071 cases and 2350 controls for miR-146a rs2910164 polymorphism, 667 cases and 1006 controls for miR-499 rs3746444 polymorphism. It was found that neither allele frequency nor genotype distribution of the two polymorphisms was associated with risk of HCC in all genetic models. Similarly, subgroup analysis in Asian population showed no associations between the two SNPs and the susceptibility to HCC.

**Conclusions/Significance:**

This meta-analysis suggests that miR-146a rs2910164 and miR-499 rs3746444 polymorphisms may not be associated with the risk of HCC, especially for Asian population. However, well-designed studies with larger sample size and more detailed data are needed to confirm these conclusions.

## Introduction

Hepatocellular carcinoma (HCC) is one of the most common malignancies worldwide [1]. Epidemiological evidence suggests that the major risk factor for HCC is chronic hepatitis B virus (HBV) and/or hepatitis C virus (HCV) infection [2]. HCC is prevalent in Middle and Western Africa and in East and Southeast Asia, especially in China where almost half of the cases and deaths occurred owing to high prevalence of infections [3]. Although HBV and HCV infections are the major causes of HCC, only a fraction of infected patients develop HCC during their lifetime, which suggests that the etiology of HCC is still not yet clarified [4]. Current studies indicated genetic factors could also contribute to the etiology of HCC [5], [6].

MicroRNAs (miRNAs) are small non-coding, single-stranded RNA molecules with typical length of 22 nucleotides. MiRNAs are involved in a wide range of physiologic and pathologic processes, including cell differentiation, proliferation, apoptosis and carcinogenesis [7], as miRNAs are involved in posttranscriptional gene expression by base pairing with target mRNAs of protein-coding genes, which can result in the destabilization and reduction in the mRNA concentration by accelerating poly (A) tail removal [7], [8].

It is reported that there is association between SNP of rs291016 in miRNA-146a and the susceptibility to HCC [9], however, other studies reported the opposite results [10], [11], [12], [13], [14]. Similarly, the conclusions of investigations about rs3746444 SNP in miRNA-499 are controversial [10], [11], [12], [15].

Since a single study with a small sample size may not be enough to detect accurate effects of these SNPs on HCC, we performed this meta-analysis to derive more comprehensive and precise estimation of the associations between the SNPs miR-146a rs2910164 and miR-499 rs11614913 and susceptibility to HCC.

## Methods

### Searching

We carried out a publication search in PubMed, Cochrane Central Register of Controlled Trials, ScienceDirect, and Chinese National Knowledge Infrastructure (CNKI) databases with the following search terms: (“miR-146a” OR “miR-499” OR “rs2910164” OR “rs3746444”) AND (“hepatocellular carcinoma” OR “liver cancer” OR “HCC”) AND (“SNP” OR “mutation” OR “variation” OR “polymorphism”)by two independent investigators (Miao Hu and Lianying Zhao, last search update: Sep 10th, 2012). Publication country and publication language were not restricted in our search. We examined reference lists manually to further identify potentially relevant studies, and contacted the corresponding authors of conference abstracts without sufficient data to get additional information by e-mail. If the author had refused to provide the data required in this meta-analysis or we had acquired no reply, the item would be excluded. All the items matching the inclusion criteria were retrieved for further examination and data extraction. Investigators include experts in hepatobiliary, surgery, biologists, epidemiologist and qualified graduate researchers. All of the investigators have received training in literature search, statistics and evidence-based medicine.

### Selection

We set the following criteria for studies recruited in our meta-analysis: (a) evaluated the associations between the SNPs miR-146a rs2910164 and/or miR-499 rs3746444 and susceptibility to HCC, (b) studied on human beings, (c)study designed as case-control, (d) There was sufficient data for the computation of odds ratios and corresponding 95% confidence intervals (ORs, 95% CIs). (e) If serial studies of the same population from the same group were reported, the latest study would be included. We assessed the methodological qualities of included studies by the description of title, author, year, country, ethnicity, source of sample, gene typing methods, the set of controls and cases, value of Hardy-Weinberg equilibrium (HWE), and factors of risk.

### Data extraction

Two investigators (Miao Hu and Lianying Zhao) screened titles, abstracts and full texts independently using a standardized screening guide. Data extraction was carried out independently after the concealment of titles, authors, journals, supporting organizations and funds to avoid investigators' bias. After the data abstraction, discrepancies and differences were resolved by consultation and discussion.

Characteristics of the enrolled studies were assigned in the structured form (Table 1), including first author's name, publication time, ethnicity, study country origin, SNP, genotyping method, total numbers of cases and controls and genotype frequencies of cases and controls.

**Table 1:** Characteristics of studies included in the meta-analysis.

Author	Juan Zhou	WonHee Kim	Hikmet Akkiz	Teng Xu	Xinwei Zhang	Yu Xiang
Year	2012	2012	2011	2008	2011	2012
Ethnicity	Asian	Asian	European	Asian	Asian	Asian
Country	China	Korea	Turkey	China	China	China
SNP	rs2910164rs3746444	rs2910164rs3746444	rs2910164rs3746444	rs2910164rs3746444	rs2910164	rs2910164
Genotyping method	PCR-RFLP	PCR-RFLP	PCR-RFLP	PCR-RFLP	PCR-RFLP	PCR-RFLP
NO Cases/controls	186/483	159/201	222/222	479/504	925/840	100/100
HWE (rs2910164)	0.056	0.190	0.384	0.119	0.149	0.506
HWE (rs3746444)	0.100	0.278	1.000	0.284		
(rs2910164)Cases CC	67	57	10	158	319	28
(rs2910164)Cases CG	86	88	75	241	450	45
(rs2910164)Cases GG	33	14	137	80	156	27
(rs2910164)Controls CC	158	74	11	197	303	33
(rs2910164)Controls CG	254	103	67	249	386	46
(rs2910164)Controls GG	71	24	144	58	151	21
(rs3746444)Cases AA	141	109	45	36		
(rs3746444)Cases AG	41	47	87	40		
(rs3746444)Cases GG	4	3	90	24		
(rs3746444)Controls AA	371	120	45	54		
(rs3746444)Controls AG	100	74	93	36		
(rs3746444)Controls AG	12	7	82	10		

Hardy-Weinberg equilibrium (HWE) was evaluated using the goodness-of-fit chi-square test. P values were presented. P<0.05 was considered representative of a departure from HWE. SNP, single nucleotide polymorphism.

The two investigators (Miao Hu and Lianying Zhao) checked the data extraction results and reached consensus on all of the data extracted. If different results were generated, they would check the data and have a discussion to come to an agreement. Two senior investigators (Surong Hu and Jingting Yang) would be invited to the discussion if disagreement still existed.

### Quantitative Data Synthesis

For each study, the departure of frequencies of miR-146a rs2910164 and miR-499 rs3746444 polymorphisms from expectation under Hardy-Weinberg equilibrium (HWE) was assessed by χ^2^ test in controls. The strength of the association between miR-146a rs2910164/miR-499 rs3746444 polymorphism and HCC risk was measured by odds ratios (ORs) with 95% confidence intervals (CIs).Pooled ORs were calculated for allele frequency comparison (miR-146a rs2910164: C versus G, miR-499 rs3746444: A versus G), additive model (miR-146a rs2910164: GC versus GG, CC versus GG, and miR-499 rs3746444: AG versus GG, AA versus GG),dominant model (miR-146a rs2910164: GC/CC versus GG, and miR-499 rs3746444: AA/AG versus GG), and recessive model (miR-146a rs2910164: CC versus GC/GG, and miR-499 rs3746444: AA versus AG/GG)respectively. The significance of pooled ORs was determined by Z-test (P<0.05 was considered statistically significant).

Statistical heterogeneity among the studies was checked by chi-square-based Q-test. A P value greater than 0.10 for Q-test indicates no significant heterogeneity existing among studies [16], so that the pooled OR was estimated by the fixed-effects model, otherwise, if the heterogeneity was significant, the random-effects model would be employed. Sensitivity analysis was carried out by deleting one single study each time to examine the influence of individual data set on the pooled ORs. Publication bias of literatures was assessed using funnel plots and Egger's test. An asymmetric plot suggests a possible publication bias and the P value of Egger's test less than 0.05 was considered representative of statistically significant publication bias [17].All of the statistical tests were performed with STATA software version 10.0 (STATA Corporation, College Station, TX, USA).

## Results

### Study Characteristics

**Figure 1:**
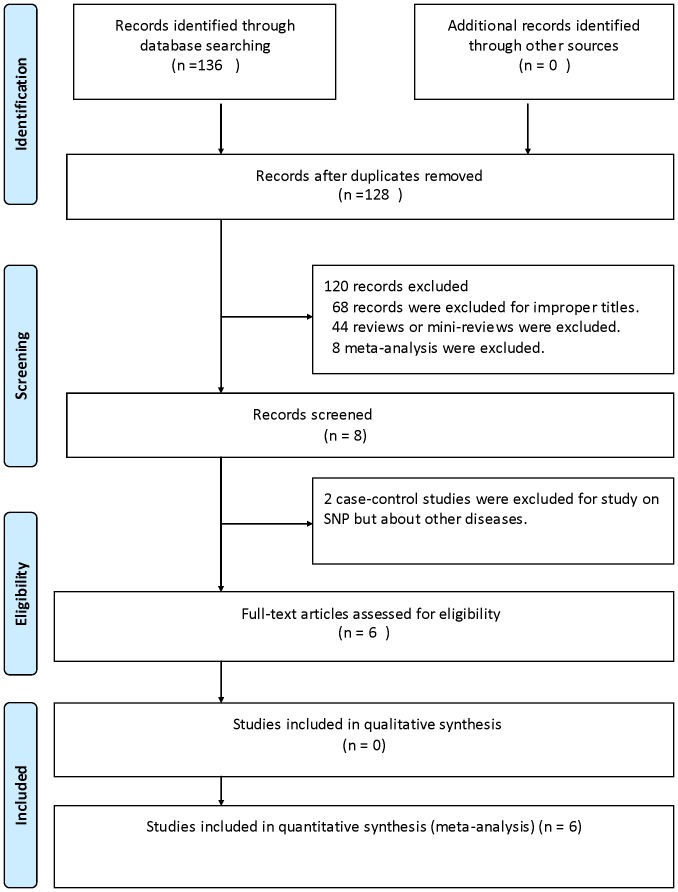
Flow diagram of study identification.

A total of 136 articles were retrieved after the first search in PubMed, ScienceDirect, Cochrane Central Register of Controlled Trials, and CNKI databases. As shown in Figure 1, after selection, 6 case-control studies fulfilled the inclusion criteria [9], [10], [11], [12], [13], [14], [15]. Characteristics of included studies are summarized in Table 1. Among all of the included studies, a total of 6 studies [9], [10], [11], [12], [13], [14] involving 2071 cases and 2350 controls for miR-146a rs2910164 and 4 studies [10], [11], [12], [15] involving 667 cases and 1006 controls for miR-499 rs3746444 were ultimately analyzed in our meta-analysis. For miR-146a rs2910164, 5 studies [9], [10], [11], [12], [14] were carried out on Asian population (Chinese and Korean population), 1 study on Caucasian population [13] (Turkish population). As for miR-499 rs3746444, there were 3 studies [10], [11], [12] on Asians (Chinese and Korean population) and 1 study on Caucasian population [15](Turkish population). All the studies included in our meta-analysis employed the same genotyping methods: polymerase chain reaction-restriction fragment length polymorphism (PCR-RFLP), except the study of Zhang XW [14]. (PIRA—PCR). The distribution of genotypes in the controls was all in agreement with HWE. MOOSE checklist was generated to provide detailed description of this meta-analysis (data available when asked).

### Meta-analysis of the Association between miR-146a rs2910164 Polymorphism and Susceptibility to HCC

The association between miR-146a rs2910164 polymorphism and susceptibility to HCC was analyzed in 6 independent studies with 2,071 cases and 2,350 controls. The result of this meta-analysis is shown in table 2. Q-test was used in all of the genetic models and showed no significant heterogeneity. Therefore, the pooled ORs were calculated using fixed-effects model. There is no significant association between miR-146a rs2910164 polymorphism and susceptibility to HCC , which could be identified in any of the genetic models (C versus G: OR = 0.94, 95% CI 0.862–1.028, P = 0.178; GC versus GG: OR = 0.977, 95% CI 0.827–1.154, P = 0.786; CC versus GG: OR = 0.874, 95% CI 0.726–1.053, P = 0.158; GC/CC versus GG: OR = 0.946, 95% CI 0.808–1.107, P = 0.486; CC versus GC/GG: OR = 0.909, 95% CI 0.797–1.036, P = 0.153).

**Table 2:** Summary of pooled ORs in the meta-analysis of SNP rs2910164.

Genetic Model	Population	Pooled OR	[95%CI]	P	P(h-t)
C versus G	overall	0.94	[0.86, 1.03]	0.178	0.274
C versus G	Asian	0.93	[0.85, 1.02]	0.124	0.236
GC versus GG	overall	0.98	[0.83, 1.15]	0.786	0.156
GC versus GG	Asian	0.94	[0.78, 1.13]	0.512	0.135
CC versus GG	overall	0.87	[0.75, 1.05]	0.158	0.219
CC versus GG	Asian	0.87	[0.72, 1.05]	0.155	0.137
DominantGC/CC versus GG	overall	0.95	[0.81,1.11]	0.486	0.124
DominantGC/CC versus GG	Asian	0.91	[0.77, 1.08]	0.284	0.111
RecessiveCC versus GC/GG	overall	0.91	[0.80, 1.04]	0.153	0.586
RecessiveCC versus GC/GG	Asian	0.91	[0.80, 1.04]	0.158	0.441

P(h-t), P-value for heterogeneity test. Random-effects model was used when the p-value for heterogeneity test < = 0.10, otherwise the fixed-effect model was used. OR, odds ratio; CI, confidence interval.

In all the six studies included, five were conducted on Asian, one study on Caucasian. In the ethnicity subgroup analysis, no association was found to be statistically significant on Asian population in any genetic model (C versus G: OR = 0.931, 95% CI 0.850–1.020, P = 0.124; GC versus GG: OR = 0.941, 95% CI 0.784–1.129, P = 0.512; CC versus GG: OR = 0.871, 95% CI 0.719–1.054, P = 0.155; GC/CC versus GG: OR = 0.910, 95% CI 0.766–1.081, P = 0.284; CC versus GC/GG: OR = 0.909, 95% CI 0.796–1.038, P = 0.158) (Table 2).

### Meta-analysis of the Association between miR-499 rs3746444 Polymorphism and Susceptibility to HCC

4 studies involving 667 cases and 1,006 controls were evaluated for the association between miR-196a2 rs11614913 polymorphism and HCC risk. Significant statistical heterogeneity was identified in the comparison of allele frequency, AA versus GG, Asian subgroup analysis of dominant model and the recessive model, so that random-effects model was used in these models, Fixed-effects model was used in other models. However, for rs3746444, none of the genetic models produced significant association between rs3746444 polymorphism and HCC risk (A versus G: OR = 0.911, 95% CI 0.770–1.078, P = 0.227; AG versus GG: OR = 0.824, 95% CI 0.585–1.161, P = 0.267; AA versus GG: OR = 0.756, 95% CI 0.518–1.102, P = 0.146; AG/AA versus GG: OR = 0.791, 95% CI 0.577–1.082, P = 0.143; AA versus AG/GG: OR = 0.955, 95% CI 0.763–1.196, P = 0.688). The results are summarized in Table 3.

**Table 3:** Summary of pooled ORs in the meta-analysis of SNP rs3746444.

Genetic Model	Population	Pooled OR	[95%CI]	P	P(h-t)
A versus G	overall	0.91	[0.77, 1.08]	0.277	0.004
A versus G	Asian	0.91	[0.73, 1.13]	0.397	0.001
AG versus GG	overall	0.82	[0.58,1.16]	0.267	0.416
AG versus GG	Asian	0.77	[0.42, 1.40]	0.385	0.251
AA versus GG	overall	0.76	[0.52,1.10]	0.146	0.04
AA versus GG	Asian	0.63	[0.36, 1.11]	0.108	0.023
DominantAG/AA versus GG	overall	0.79	[0.58,1.08]	0.143	0.107
DominantAG/AA versus GG	Asian	0.66	[0.38, 1.15]	0.143	0.062
RecessiveAA versus AG/GG	overall	0.96	[0.76,1.20]	0.688	0.024
RecessiveAA versus AG/GG	Asian	0.96	[0.74,1.24]	0.743	0.009

P(h-t), P-value for heterogeneity test. Random-effects model was used when the p-value for heterogeneity test < = 0.10, otherwise the fixed-effect model was used. OR, odds ratio; CI, confidence interval.

Similarly, subgroup analysis showed no significant association between SNP rs3746444 and susceptibility to HCC in Asian population (A versus G: OR = 0.911, 95% CI 0.734–1.131, P = 0.397; AG versus GG: OR = 0.767, 95% CI 0.422–1.395, P = 0.385; AA versus GG: OR = 0.630, 95% CI 0.358–1.106, P = 0.108; AG/AA versus GG: OR = 0.663, 95% CI 0.382–1.150, P = 0.143; CC versus TC/TT: OR = 0.958, 95% CI 0.740–1.239, P = 0.743)(Table 3).

### Publication Bias

**Figure 2:**
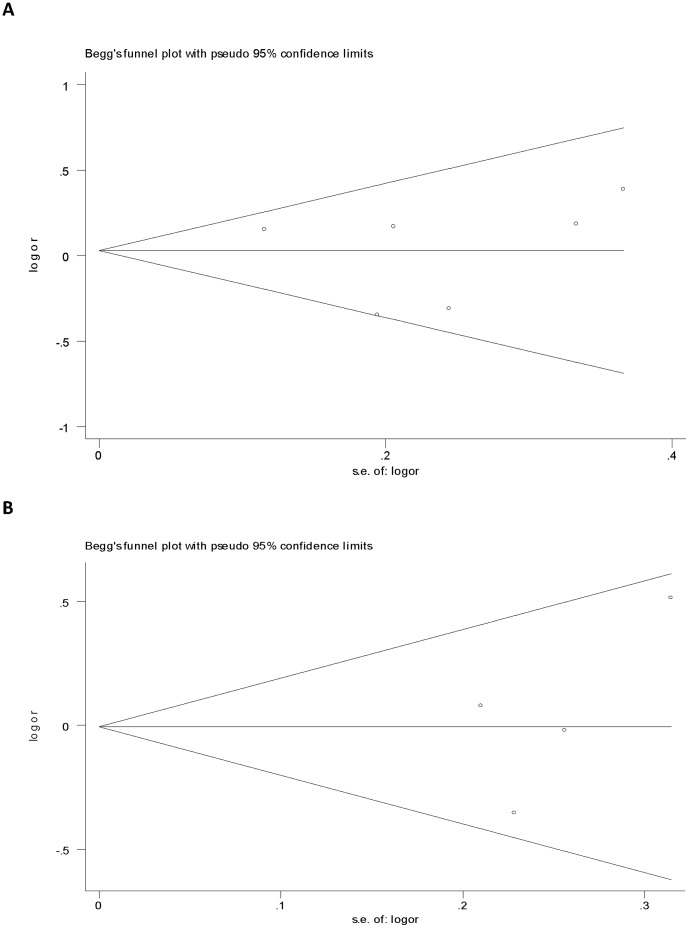
Begg's funnel plot of publication bias for rs11614913 and rs3746444. A. Begg's funnel plot for rs11614913(C versus G). B. Begg's funnel plot for rs3746444 (A versus G). Log OR is plotted versus standard error of Log OR for each included study. Every circle dot represents a separate study for the indicated association by allele contrast.

Funnel plot and Egger's test were performed to assess the publication bias of the literature (Figure 2). Symmetrical funnel plots were obtained in both of the SNPs tested in all of the models. Egger's test further confirmed the absence of publication bias in this meta-analysis (P<0.05). No evidence of publication bias was observed in any comparison model.

### Sensitivity Analysis

We deleted one single study from the overall pooled analysis each time to check the influence of the removed data set to the pooled ORs. No study was observed to change the homogeneity in heterozygote comparison.

## Discussion

The research on microRNAs has provided us a new approach to understand the molecular mechanism of HCC and a new therapy to treat HCC [18]. As every miRNA molecule could have hundreds of target genes to modulate, even a single nucleotide mutation in miRNAs could have profound impacts on the expression of numerous genes including oncogenes and anti-oncogenes, they would finally influence individual's susceptibility to cancer [7]. Indeed, the rs3746444 SNP in miRNA-499 and rs291016 SNP in miRNA-146a have been reported to be associated with the susceptibility to squamous cell carcinoma of the head and neck [19], and SNP in miRNA-146a contributes to the genetic predisposition to papillary thyroid carcinoma [20] .

Recently, numerous reports have revealed that SNPs in miRNA-coding region may be involved in the process of hepatocarcinogenesis and contribute to the development of HCC [9], [10], [11], [12], [13], [14], [21], [22].

### For microRNA146a rs2910164

miR-146a rs2910164 is located in the stem region opposite to the mature miR-146a sequence. This C>G polymorphism results in a change from C: U pair to G: U mismatch in the stem structure of miR-146a precursor. Compared with C allele, The G allele gene could lead to more efficient inhibition of target genes including IL-1 receptor-associated kinase 1 (IRAK1), TNF receptor-associated factor 6 (TRAF6) and papillary thyroid carcinoma 1 gene (PTC1) [20]. Several case-control studies have investigated the association between miR-146a rs2910164 polymorphism and risks of various cancers [23], [24], [25]. Moreover, SNPs rs2910164 in miR-146a was thought to contribute to modified HCC risks [9], [10], [11], [12], [13], [14], but the conclusions are controversial and dubious because of the limited sample size, potential selection bias, and other unsuspected reasons.

To study the relationship between the miR-146a rs2910164 polymorphism and HCC, we made a meta-analysis including 6 case-control studies with 2071 cases and 2350 controls. Our results did not support a genetic association between rs2910164 and susceptibility to HCC. Neither allele frequency nor genotype distribution was significantly associated with susceptibility to HCC.

Considering the potential relationship between the gene polymorphisms in different ethnic groups and the HCC risk, we further made the subgroup analysis on Asian population to explore the association between rs2910164 and the risk of HCC. As a result, no association was observed even in the same ethnic population.

Furthermore, deficiency of detailed but important information of both cases and controls such as virus infection, alcohol consumption, age and gender has inhibited us from exploring the relationships between rs2910164 polymorphism and HCC risk more thoroughly. Considering the important biological function of miR-146a in tumorigenesis [26], [27], [28], the conclusion that miR-146a rs2910164 polymorphism has no role in HCC development is premature due to limited number of studies included in this study.

### For MicroRNA499 rs3746444

Another SNP shown to have potential relationship to the risk for HCC is rs3746444 in miR-499. miR-499 was reported to play a role of mediator in a wide spectrum of biological processes, such as cellular senescence, apoptosis, immune response, tumorigenesis and metastasis [29], [30], [31].And more molecular epidemiological studies have investigated the association between the miR-499 rs3746444 polymorphism and cancer [29], [30]. Indeed, recent studies have investigated the association of miR-499 rs11614913 polymorphism with HCC risk [10], [11], [12], [15], but the conclusions are inconsistent.

To study the relationship between the miR-499 rs3746444 polymorphism and HCC, we made a meta-analysis including 4 case-control studies with 667cases and 1006 controls. As a result, our study did not support a genetic association between rs3746444 and susceptibility to HCC. Neither allele frequency nor genotype distribution was significantly associated with susceptibility to HCC. Similar to the miR-146a rs2910164, subgroup analysis on Asian population also showed no association of miR-499 rs3746444 polymorphism with susceptibility to HCC. Sensitivity analysis found no significant influence of any single study on pooled ORs, which indicated the stability of this meta-analysis was acceptable.

### Comparisons with other Meta-analyses

Firstly, although several meta-analyses have reported associations between these two SNPs and susceptibility to various cancers [32], [33], [34], [35], [36], [37], [38], [39], most of these meta-analyses reported statistically significant associations between the two SNPs and susceptibility to cancer without pre-specified tissue origin, and they mainly focused on the clinical heterogeneity brought by inherent difference among cancers from various tissue origins, which would limit the reliability and accuracy of the conclusions about the relationship between miRNAs and HCC.

Secondly, for rs2910164 in microRNA146a, In Wang's study [40], he studied the association between this SNP and HCC. But we found that he missed the study finished by Won Hee Kim [11] about the relationship between microRNA146a in Korean population and HCC. This would decrease the credibility and accuracy of his meta-analysis although we and Wang [40] reached similar conclusion.

Thirdly, for rs3746444 SNP in miRNA-499, by the date of Sep 10^th^ 2012, several meta-analyses have investigated the relationship between this SNP and risk of cancer [32], [33], [34], but one of them has not studied the association between this SNP and HCC [32] ;and for the others of them, several association studies with eligible data were left out in their subgroup analysis on HCC [33], [34], this would reduce the reliability of their conclusions. We have not found any other research that focused on the relationship between SNP of miRNA-499 and susceptibility of HCC to date.

In our present meta-analysis, we finished comprehensive literature search in multiple databases as many as possible no matter what the publication languages, dates, ethnicities and other factors that would limit data collection are. Quantitative data synthesis were conducted in allele frequency, additive model, dominant model and recessive model and we found none of the SNPs was associated with the risk of HCC with more powerful evidence compared with previous similar meta-analyses.

### Limitations

To the best of our knowledge, this is the first meta-analysis evaluating the potential association between two common polymorphisms rs2910164 in miR-146a and rs3746444 in miRNA-499 and susceptibility to HCC. However, limitations of this meta-analysis should be discussed as they may affect the interpretation of the results.

Firstly, the sample size is relatively small although we have searched as many eligible literatures as possible, larger sample size and multiple random-control tests are still needed to detect possible minor effects of miR-146a rs2910164 and rs3746444 in miRNA-499 polymorphisms on susceptibility to HCC.

Secondly, lack of available data prevented an adjustment for subgroup factors including alcohol consumption, age, and gender, etc. These factors may become potential determinants to influence the evaluation of the associations between SNPs and susceptibility to HCC by interacting with genetic factors.

Thirdly, there was no study on African population in the meta-analysis, and the study on Caucasian is not enough, limitation of the ethnic population enrolled makes the interpretation of the association between the two SNPs and susceptibility to HCC should be more cautious.

Finally, it has been generally accepted that HBV is one of the most important factors of HCC [4], however, we could not carry out our stratified study analysis on HBV infection because of the limited data about HBV infection status including virus titer, the period of HBV infection and CHILD classification. Furthermore, there must be other latent factors which would have potential effects on the occurrence and development of HCC, but they have not been realized yet. Lack of this information has prevented us from exploring the relationship further. It should be acknowledged when interpreting the results of the meta-analysis.

In conclusion, this meta-analysis provides more evidence that miR-146a rs2910164 and miR-499 rs3746444 polymorphisms may not be associated with the risk of HCC, especially for Asian population. Well-designed studies with larger sample size and more ethnic groups are of great value to clarify these findings. Moreover, combination genetic variation together with factors such as HBV/HCV infection status, gender, age, and alcohol consumption exposures should also be considered in future.

## Supporting Information

Table S1 Checklist.
(DOC)

Table S2 Moose test.
(DOCX)

## References

[1] Nguyen MH, Keeffe EB (2004) Epidemiology and treatment outcomes of patients with chronic hepatitis C and genotypes 4 to 9. Rev Gastroenterol Disord 4 Suppl 1: S14–21. 15184820

[2] Bouchard MJ, Navas-Martin S (2011) Hepatitis B and C virus hepatocarcinogenesis: lessons learned and future challenges. Cancer Lett 305: 123–143. 21168955 10.1016/j.canlet.2010.11.014 PMC3071446

[3] El-Serag HB, Rudolph KL (2007) Hepatocellular carcinoma: epidemiology and molecular carcinogenesis. Gastroenterology 132: 2557–2576. 17570226 10.1053/j.gastro.2007.04.061

[4] El-Serag HB (2011) Hepatocellular carcinoma. N Engl J Med 365: 1118–1127. 21992124 10.1056/NEJMra1001683

[5] Pogribny IP, Rusyn I (2012) Role of epigenetic aberrations in the development and progression of human hepatocellular carcinoma. Cancer Lett 10.1016/j.canlet.2012.01.038 PMC3971756 22306342

[6] Hagymasi K, Tulassay Z (2008) [Epidemiology, risk factors and molecular pathogenesis of primary liver cancer]. Orv Hetil 149: 541–548. 18343770 10.1556/OH.2008.28313

[7] Bartel DP MicroRNAs: genomics, biogenesis, mechanism, and function. [Review] [125 refs]. Cell 116: 281–297. 14744438 10.1016/s0092-8674(04)00045-5

[8] Bartel B MicroRNAs directing siRNA biogenesis. Nat Struct Mol Biol 12: 569–571. 15999111 10.1038/nsmb0705-569

[9] Xu T, Zhu Y, Wei QK, Yuan Y, Zhou F, et al (2008) A functional polymorphism in the miR-146a gene is associated with the risk for hepatocellular carcinoma. Carcinogenesis 29: 2126–2131. 18711148 10.1093/carcin/bgn195

[10] Zhou J, Lv R, Song X, Li D, Hu X, et al (2012) Association between two genetic variants in miRNA and primary liver cancer risk in the Chinese population. DNA Cell Biol 31: 524–530. 21861697 10.1089/dna.2011.1340 PMC3322400

[11] Kim WH, Min KT, Jeon YJ, Kwon CI, Ko KH, et al (2012) Association study of microRNA polymorphisms with hepatocellular carcinoma in Korean population. Gene 504: 92–97. 22583825 10.1016/j.gene.2012.05.014

[12] Xiang Y, Fan S, Cao J, Huang S, Zhang LP (2012) Association of the microRNA-499 variants with susceptibility to hepatocellular carcinoma in a Chinese population. Mol Biol Rep 39: 7019–7023. 22311030 10.1007/s11033-012-1532-0

[13] Akkiz H, Bayram S, Bekar A, Akgollu E, Uskudar O, et al (2011) No association of pre-microRNA-146a rs2910164 polymorphism and risk of hepatocellular carcinoma development in Turkish population: a case-control study. Gene 486: 104–109. 21807077 10.1016/j.gene.2011.07.006

[14] Zhang XW, Pan SD, Feng YL, Liu JB, Dong J, et al (2011) [Relationship between genetic polymorphism in microRNAs precursor and genetic predisposition of hepatocellular carcinoma]. Zhonghua Yu Fang Yi Xue Za Zhi 45: 239–243. 21624236

[15] Akkiz H, Bayram S, Bekar A, Akgollu E, Uskudar O (2011) Genetic variation in the microRNA-499 gene and hepatocellular carcinoma risk in a Turkish population: lack of any association in a case-control study. Asian Pac J Cancer Prev 12: 3107–3112. 22393998

[16] Lau J, Ioannidis JP, Schmid CH Quantitative synthesis in systematic reviews. Ann Intern Med 127: 820–826. 9382404 10.7326/0003-4819-127-9-199711010-00008

[17] Egger M, Davey Smith G, Schneider M, Minder C Bias in meta-analysis detected by a simple, graphical test.[see comment]. BMJ 315: 629–634. 10.1136/bmj.315.7109.629 PMC2127453 9310563

[18] Law PT, Wong N (2011) Emerging roles of microRNA in the intracellular signaling networks of hepatocellular carcinoma. J Gastroenterol Hepatol 26: 437–449. 21332540 10.1111/j.1440-1746.2010.06512.x

[19] Liu Z, Li G, Wei S, Niu J, El-Naggar AK, et al (2010) Genetic variants in selected pre-microRNA genes and the risk of squamous cell carcinoma of the head and neck. Cancer 116: 4753–4760. 20549817 10.1002/cncr.25323 PMC3030480

[20] Jazdzewski K, Murray EL, Franssila K, Jarzab B, Schoenberg DR, et al (2008) Common SNP in pre-miR-146a decreases mature miR expression and predisposes to papillary thyroid carcinoma. Proc Natl Acad Sci U S A 105: 7269–7274. 18474871 10.1073/pnas.0802682105 PMC2438239

[21] Braconi C, Henry JC, Kogure T, Schmittgen T, Patel T (2011) The role of microRNAs in human liver cancers. Semin Oncol 38: 752–763. 22082761 10.1053/j.seminoncol.2011.08.001 PMC3928803

[22] Lovat F, Valeri N, Croce CM MicroRNAs in the Pathogenesis of Cancer. Semin Oncol 38: 724–733. 22082758 10.1053/j.seminoncol.2011.08.006

[23] Mittal RD, Gangwar R, George GP, Mittal T, Kapoor R Investigative Role of Pre-MicroRNAs in Bladder Cancer Patients: A Case-Control Study in North India. DNA Cell Biol 30: 401–406. 21345130 10.1089/dna.2010.1159

[24] Pastrello C, Polesel J, Della Puppa L, Viel A, Maestro R Association between hsa-mir-146a genotype and tumor age-of-onset in BRCA1/BRCA2-negative familial breast and ovarian cancer patients. Carcinogenesis 31: 2124–2126. 10.1093/carcin/bgq184 20810544

[25] Srivastava K, Srivastava A, Mittal B (2010) Common genetic variants in pre-microRNAs and risk of gallbladder cancer in North Indian population. J Hum Genet 55: 495–499. 20520619 10.1038/jhg.2010.54

[26] Bhaumik D, Scott GK, Schokrpur S, Patil CK, Campisi J, et al (2008) Expression of microRNA-146 suppresses NF-kappaB activity with reduction of metastatic potential in breast cancer cells. Oncogene 27: 5643–5647. 18504431 10.1038/onc.2008.171 PMC2811234

[27] Hurst DR, Edmonds MD, Scott GK, Benz CC, Vaidya KS, et al (2009) Breast cancer metastasis suppressor 1 up-regulates miR-146, which suppresses breast cancer metastasis. Cancer Res 69: 1279–1283. 19190326 10.1158/0008-5472.CAN-08-3559 PMC2754225

[28] Li Y, Vandenboom TG 2nd, Wang Z, Kong D, Ali S, et al (2010) miR-146a suppresses invasion of pancreatic cancer cells. Cancer Res 70: 1486–1495. 20124483 10.1158/0008-5472.CAN-09-2792 PMC2978025

[29] Lafferty-Whyte K, Cairney CJ, Jamieson NB, Oien KA, Keith WN (2009) Pathway analysis of senescence-associated miRNA targets reveals common processes to different senescence induction mechanisms. Biochim Biophys Acta 1792: 341–352. 19419692 10.1016/j.bbadis.2009.02.003

[30] Wang JX, Jiao JQ, Li Q, Long B, Wang K, et al (2011) miR-499 regulates mitochondrial dynamics by targeting calcineurin and dynamin-related protein-1. Nat Med 17: 71–78. 21186368 10.1038/nm.2282

[31] Hu Z, Chen X, Zhao Y, Tian T, Jin G, et al (2010) Serum microRNA signatures identified in a genome-wide serum microRNA expression profiling predict survival of non-small-cell lung cancer. J Clin Oncol 28: 1721–1726. 20194856 10.1200/JCO.2009.24.9342

[32] Wang L, Qian S, Zhi H, Zhang Y, Wang B, et al (2012) The association between hsa-miR-499 T>C polymorphism and cancer risk: a meta-analysis. Gene 508: 9–14. 22903035 10.1016/j.gene.2012.08.005

[33] Wang Y, Yang B, Ren X (2012) Hsa-miR-499 polymorphism (rs3746444) and cancer risk: a meta-analysis of 17 case-control studies. Gene 509: 267–272. 22922391 10.1016/j.gene.2012.08.008

[34] Wang F, Sun G, Zou Y, Li Y, Hao L, et al (2012) Association of microRNA-499 rs3746444 polymorphism with cancer risk: evidence from 7188 cases and 8548 controls. PLoS One 7: e45042. 22970328 10.1371/journal.pone.0045042 PMC3438197

[35] Wang J, Bi J, Liu X, Li K, Di J, et al (2012) Has-miR-146a polymorphism (rs2910164) and cancer risk: a meta-analysis of 19 case-control studies. Mol Biol Rep 39: 4571–4579. 21947843 10.1007/s11033-011-1247-7

[36] Xu W, Xu J, Liu S, Chen B, Wang X, et al (2011) Effects of common polymorphisms rs11614913 in miR-196a2 and rs2910164 in miR-146a on cancer susceptibility: a meta-analysis. PLoS One 6: e20471. 21637771 10.1371/journal.pone.0020471 PMC3102728

[37] Tian T, Xu Y, Dai J, Wu J, Shen H, et al (2010) Functional polymorphisms in two pre-microRNAs and cancer risk: a meta-analysis. Int J Mol Epidemiol Genet 1: 358–366. 21532845 PMC3076781

[38] Qiu LX, He J, Wang MY, Zhang RX, Shi TY, et al (2011) The association between common genetic variant of microRNA-146a and cancer susceptibility. Cytokine 56: 695–698. 21978540 10.1016/j.cyto.2011.09.001

[39] Wang J, Wang Q, Liu H, Shao N, Tan B, et al (2012) The association of miR-146a rs2910164 and miR-196a2 rs11614913 polymorphisms with cancer risk: a meta-analysis of 32 studies. Mutagenesis 27: 779–788. 22952151 10.1093/mutage/ges052

[40] Wang Z, Cao Y, Jiang C, Yang G, Wu J, et al (2012) Lack of association of two common polymorphisms rs2910164 and rs11614913 with susceptibility to hepatocellular carcinoma: a meta-analysis. PLoS One 7: e40039. 22768213 10.1371/journal.pone.0040039 PMC3386926

