# Supplementary material for: The Association between Two Common Polymorphisms in MicroRNAs and Hepatocellular Carcinoma Risk in Asian Population
Source: PLoS One. 2013 Feb 20;8(2):e57012. doi: 10.1371/journal.pone.0057012 (PMC3577770; doi:10.1371/journal.pone.0057012)
Supplement: Table S2 — Moose test. (DOCX) [file pone.0057012.s002.docx]

**MOOSE Checklist**

| **Criteria** | | **Brief description of how the criteria were handled in the meta-analysis** |
| --- | --- | --- |
| **Reporting of background should include** | |  |
| √ | Problem definition | miRNAs play crucial roles in various biological processes and may be associated with the risk for hepatocellular carcinoma (HCC). The association between two common single nucleotide polymorphisms (SNPs) rs2910164 in miR-146a and rs3746444 in miR-499 and susceptibility to HCC were inconsistent in previous studies. |
| √ | Hypothesis statement | SNPs rs2910164 and rs3746444 may affect susceptibility to HCC. |
| √ | Description of study outcomes | Hepatocellular carcinoma |
| √ | Type of exposure or intervention used | GC, CC, GC/CC genotypes or C allele in miR-146a  AG, GG, AG/GG genotypes or G allele in miR-499 |
| √ | Type of study designs used | Case-control studies. |
| √ | Study population | No restriction |
| **Reporting of search strategy should include** | |  |
| √ | Qualifications of searchers | Investigators include experts in hepatobiliary surgery, biologists and qualified graduate students. All of the investigators have received training in literature research, statistics and evidence-based medicine. |
| √ | Search strategy, including time period included in the synthesis and keywords | Publication time was restricted until 10th Sep 2012. We searched all available literatures in the following databases: PubMed 1966 –Sep 2012, CNKI 1915 – Sep 2012, Cochrane Central Register of Controlled Trials, ScienceDirect, Key words: miR-146a, miR-499, rs2910164, rs3746444, hepatocellular carcinoma, liver cancer, and HCC |
| √ | Databases and registries searched | PubMed , CNKI, Cochrane Central Register of Controlled Trials, ScienceDirect, |
| √ | Search software used, name and version, including special features | We did not employ any search software. |
|  | Use of hand searching | Reference lists of retrieved fulltexts were searched manually |
| √ | List of citations located and those excluded, including justifications | Literature search and selection process are outlined in the flow diagram. The reasons for exclusion were listed in the flow diagram. The citation list is available upon request. |
|  | Method of addressing articles published in languages other than English | Language was not restricted. |
| √ | Method of handling abstracts and unpublished studies | We first examined if overlap existed and excluded overlapped studies. We tried to contact authors for unpublished data. If authors were not willing to provide data or the requests were not responded, we excluded the study. |
| √ | Description of any contact with authors | We contacted researchers who conducted related studies to obtain detailed genotype frequencies. However, none of the researchers agreed to provide the data we needed. |
| **Reporting of methods should include** | |  |
| √ | Description of relevance or appropriateness of studies assembled for assessing the hypothesis to be tested | Detailed inclusion and exclusion criteria were described in the methods. |
| √ | Rationale for the selection and coding of data | Data extracted from each of the studies were relevant to the population characteristics, study design, genotyping methods, genotypes, outcome, and possible effect modifiers of the association. |
| √ | Assessment of confounding | Subgroup analysis in ethnicity was performed and we conducted sensitivity analysis by deleting a single study one by one for each time. |
| √ | Assessment of study quality, including blinding of quality assessors; stratification or regression on possible predictors of study results | We assessed the methodological qualities of included studies by the description of study population, detailed genotyping methods, the set of controls and cases and related statistical methods. We carried out sensitivity analysis and none of the studies was identified to have major influence on the result of our meta-analysis. |
| √ | Assessment of heterogeneity | Statistical heterogeneity among the studies was checked by chi-square-based *Q*-test. A *P*-value greater than 0.10 for *Q*-test indicates no significant heterogeneity existed among studies |
| √ | Description of statistical methods in sufficient detail to be replicated | Methods of heterogeneity test, quantitative synthesis, assessments of publication bias, sensitivity analyses are reported in detail in the methods section. |
| √ | Provision of appropriate tables and graphics | We provided flow chart to explain literature searching and selection (Figure 1); funnel plots for the explanation of publication bias (Figure 2), study characteristics and allele/genotype frequencies (Table 1); pooled analysis of rs2910164 and pooled analysis of rs3746444 (Table 2 and Table 3). |
| **Reporting of results should include** | |  |
|  | Graph summarizing individual study estimates and overall estimate | We provide individual pooled ORs, 95% CIs and *P*-values for *Z* test were summarized in Table 2 and Table 3. The graphs of results of sensitivity analysis could be provided when asked |
| √ | Table giving descriptive information for each study included | Descriptive information for each study included was provided in Table 1. |
| √ | Results of sensitivity testing | The results of sensitivity analysis were described in results section. The graphs of results of sensitivity analysis could be provided when asked |
| √ | Indication of statistical uncertainty of findings | The results of heterogeneity test, pooled ORs, 95% confidence intervals and *P* value for *Z* test were presented with all pooled analyses. Results of sensitivity analysis were described and explained. |
| **Reporting of discussion should include** | |  |
| √ | Quantitative assessment of bias | We evaluated the publication bias by funnel plots and egger’s test. No significant publication bias was detected. |
| √ | Justification for exclusion | Only case-control studies which meet our inclusion criteria were included. Reviews, meta-analysis, studies on other cancers than HCC and other irrelevant studies which were inconsistent to the inclusion criteria were excluded. |
| √ | Assessment of quality of included studies | We discussed the characteristics and limitations of included studies. |
| **Reporting of conclusions should include** | |  |
| √ | Consideration of alternative explanations for observed results | We discussed that potential unmeasured confounders and explained the limitations of this meta-analysis. We reminded readers that caution should be made when interpreting this meta-analysis. |
| √ | Generalization of the conclusions | This meta-analysis suggests that two common SNPs rs2910164 and rs3746444 may not be associated with the risk of HCC, especially for Asian population. |
| √ | Guidelines for future research | Further well-designed studies with larger sample size and more ethnic groups are needed to further validate the association. |
| √ | Disclosure of funding source | This study was an independent research |
